# Multi-Fiber-Reinforced Composites for the Coronoradicular Reconstruction of Premolar Teeth: A Finite Element Analysis

**DOI:** 10.1155/2018/4302607

**Published:** 2018-06-07

**Authors:** Raphaël Richert, Philip Robinson, Gilbert Viguie, Jean-Christophe Farges, Maxime Ducret

**Affiliations:** ^1^Université de Lyon, Université Lyon 1, Faculté d'Odontologie, Lyon, France; ^2^Hospices Civils de Lyon, Service de Consultations et Traitements Dentaires, Lyon, France; ^3^Hospices Civils de Lyon, DRCI, Lyon, France; ^4^Laboratoire de Biologie Tissulaire et Ingénierie Thérapeutique, UMR5305 CNRS/Université Lyon 1, UMS3444 BioSciences Gerland-Lyon Sud, Lyon, France

## Abstract

A coronoradicular reconstruction (CRR) has conventionally used a metallic inlay core (MIC) or a single-fiber-reinforced composite (sFRC) but extensive dentin removal can lead to root fracture. We propose herein a multi-fiber-reinforced composite (mFRC) based on a bundle of thin flexible fibers that can be adapted to the root anatomy without removing additional dentin. The aim of this study was to compare the mechanical behavior of the root reconstructed with mFRC, MIC, or sFRC using a finite element analysis (FEA). Models with or without a ferrule effect were created using Autodesk^©^ software and divided into four parts: root, post, bonding composite or cement, and zirconia crown. For both models, extreme stress values (ESV), stress distribution, and risk of fracture were calculated for an oblique force (45°) of 100 N applied to the top of the buccal cusp. Results indicated that mFRC and mFRCG present a lower risk of fracture of the root and of the CRR without ferrule and thus could be valuable alternatives for premolar CRR. Further studies are necessary to evaluate the clinical success of these CRR.

## 1. Introduction

Coronoradicular reconstruction (CRR) is classically recommended when an endodontically treated tooth cannot be restored using coronal reconstruction [1, 2]. This strategy allows replacement of lost dentin, stabilizes the crown, and ensures resistance against cervical tooth fracture [3]. Currently, CRR are performed directly, using a single post in a fiber-reinforced composite (sFRC) or, indirectly, using the traditional metallic inlay core (MIC) [4, 5]. However, sFRC standard post placement and MIC post impression often imply extensive removal of the root dentin, which is a major drawback, since tissue preservation including the ferrule effect (FE) is strongly associated with the survival of endodontically treated teeth [6–8]. Moreover, MIC and sFRC present a greater elastic modulus than that of dentin, which also increases the risk of tooth fracture [9]. Clearly, important tissue removal and differential mechanical behavior weaken the root and lead to low tooth survival [10–14]. Furthermore, reconstructed premolars have a lower survival rate due to smaller crowns and lateral occlusal forces [15]. Alternative CRR strategies have therefore been investigated without clear consensus [16–19]. A new kind of FRC, based on a bundle of fibers bonded in the root canal, is proposed in the present study. This multi-fiber-reinforced composite (mFRC) can be adapted to the root anatomy without additional dentin removal after root canal treatment. Furthermore, adding a gutta-percha point in the center of the fiber bundle (mFRCG) is possible, which, in case of root reinfection, enables easier reintervention in the root than when a metal or single fiber post is used.

Before performing a clinical trial that will necessitate a large number of patients to deal with anatomical and clinical variations, valid comparison of these different CRR is required. Finite element analysis (FEA) has been widely used to evaluate mechanical behavior of CRR in dentistry [20–23], yet, to the best of our knowledge, there is no published study on a CRR using either mFRC or mFRCG. Our aim was therefore to compare, using FEA, the risk of root fracture of mFRC and mFRCG with that of MIC and sFRC.

## 2. Materials And Methods

### 2.1. Model Construction

The model was constructed using professional software (Autodesk Inc., Inventor, San Rafael, CA, US) to represent a premolar tooth endodontically treated and supporting a CRR and a zirconia crown. Four parts (root, post(s), bonding composite or sealing cement, and crown) were modeled independently and then matched together using the software to generate a complete 3D model.

**Figure 1:**
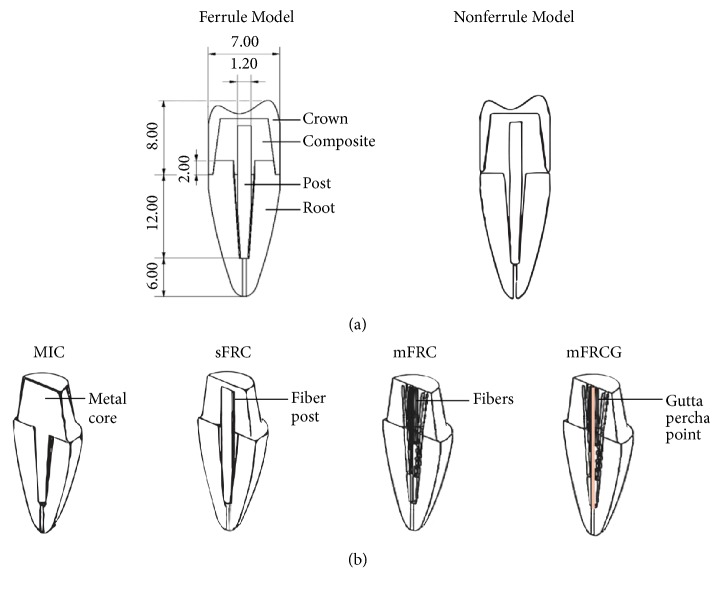
Representative figures of the models used in the study. Profile views of the ferrule and nonferrule models (a). Three-quarter view of the four different coronoradicular reconstructions (CRR): MIC (metal inlay core), single-fiber-reinforced composite (sFRC), multi-fiber-reinforced composite (mFRC), and multi-fiber-reinforced composite with gutta (mFRCG) for nonferrule models (b). Dimensions are in mm.

Eight models were generated with or without a ferrule and with four different CRRs: MIC, sFRC, mFRC, and mFRCG. Dimensions of the four parts of the 3D model were chosen according to the literature [21–25]. The ferrule effect was a 2 mm high wall that was at least 1 mm thick. The 3D model measured 26 mm in length and 7 mm in width, with an 8 mm long zirconia crown and an 18 mm long root. The post occupied two-thirds of the length of the root and was 1.2 mm in diameter (Figure 1(a)). In the MIC models, inlay cores were sealed using 0.1 mm thick cement. sFRC models were created using a standard cylindrical-conical post that was 1.2 mm in diameter and 16 mm in length bonded with composite. FRC models were designed using a bonding composite that was reinforced by 14 flexible fibers bonded in the root at different levels (Figure 1(b)). The area between the post and the root was named the interface and corresponded to sealing cement or bonding composite according to the model. FRCG models included a central gutta-percha point replacing the central fiber. The gutta-percha point had the same dimensions as the central fiber.

### 2.2. Material Properties And Mesh

A static structural analysis was performed to calculate extreme stress values (ESV) and the stress distribution on the models. All structures were assumed to be linearly elastic, homogeneous, and isotropic [26]. Ideal adherence was assumed between structures (zirconia with cement, cement with core, core with post, post with cement, and cement with dentin interfaces). The Poisson ratio (n) and modulus of elasticity (E) of the oral tissue and crown material were determined from the literature [24–27] and are given in Table 1. All models were meshed by about 110 000 elements and 200 000 nodes according to a convergence study [28].

### 2.3. Load And External Conditions

All models received an oblique force of 100 N at 45 degrees at the top of the buccal cusp to simulate masticatory forces. External surfaces of the tooth were supposed to be clamped without freedom to rotate in any direction to model bone anchorage. von Mises stresses and risk of fracture (ROF) were calculated after loading on each part of the model. The stress on the buccal side represents compressive stress, whereas the stress on the palatal side corresponds to traction stress. The ROF was calculated by dividing the maximal principal stress in each material by its tensile strength. When the ROF value was lower than 1, it was considered low [25].

## 3. Results

### 3.1. Extreme Stress Value

ESV were lower with ferrule than without ferrule, and they were always maximal on the root irrespective of the presence of ferrule. ESV for mFRC and mFRCG were close for the model with ferrule and for the model without ferrule (Table 2).

With ferrule, the ESV on the root for all types of reconstruction were close (less than 5 MPa difference (Table 2)); there was therefore a similar ROF for the root among the types of reconstruction tested (Table 3). The ESV on the CRR and at the interface were highest for MIC, intermediate for mFRC and mFRCG, and lowest for sFRC (Table 2). The ROF of CRR was lower for mFRC and mFRCG than for MIC and sFRC (Table 3).

Without ferrule, the ESV on the root was highest for sFRC (Table 2); the ROF of the root was therefore highest for sFRC (Table 3). The ESV on the CRR was highest for MIC, intermediate for mFRC and mFRCG, and lowest for sFRC. The ESV at the interface was highest for MIC; for the other types of reconstruction it was lower and relatively close (Table 2). The ROF of the root was much higher for sFRC than for other types of reconstructions tested. The ROF of the CRR was lower for mFRC and mFRCG than for sFRC (Table 3).

### 3.2. Stress Distribution

**Figure 2:**
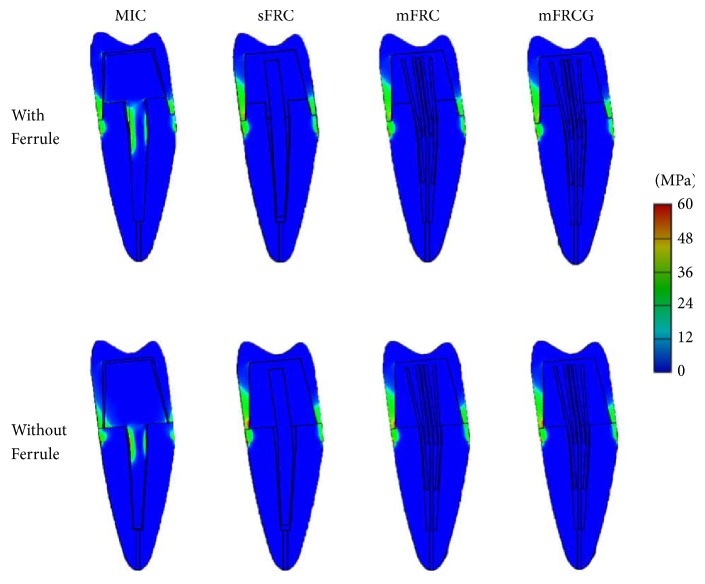
Distribution of von Mises stress (MPa) of each component of ferrule and nonferrule models revealing differences between MIC (metal inlay core) and other reconstructions: single-fiber-reinforced composite (sFRC), multi-fiber-reinforced composite (mFRC), and multi-fiber-reinforced composite with gutta (mFRCG).

**Figure 3:**
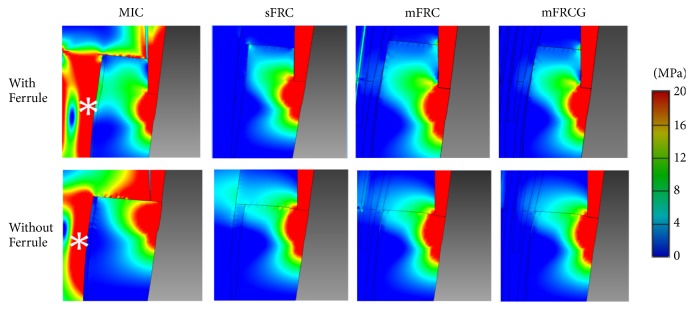
Enlarged view of post/root interface revealing higher stress around the post for MIC and hence a risk of severe horizontal root fracture. The asterisk indicates the zone of highest stress on the post of MIC model.

Stress distribution for models with ferrule was close to that of models without ferrule (Figure 2). The stress on MIC models was maximal at the buccal side of the peripheral margin and at the middle of the post and decreased progressively between the post and the peripheral margin (Figure 3). Stress distribution of sFRC, mFRC, and mFRCG was close. For these, the stress was maximal at the peripheral side of the crown margin and decreased progressively to the center of the CRR (Figure 3).

## 4. Discussion

Considering both the ESV and stress distribution data, the present study indicates that the risk of root fracture in mFRC was lower than in reconstruction using sFRC and MIC in the absence of ferrule and that it was close to that of other reconstructions investigated in the presence of ferrule.

sFRC is a widely reported alternative to MIC, particularly for fragile premolars [29–31]. Herein, although the level of stress on the root and the risk of root fracture were close in the ferrule model for the different reconstructions tested, they were notably higher for sFRC in the nonferrule model. A similar finding was reported by Mahmoudi et al. who show that, without ferrule, the ESV on the root was higher for sFRC than for MIC [19]. Similarly, Santos-Filho et al. found that the mean resistance to root fracture was significantly lower for sFRC (918 N) than for MIC (1026 N) [27]. These data suggest that sFRC are not indicated for severely damaged roots in which a ferrule is not possible.

MIC does not seem to be a particularly valuable option, since it had the highest level of stress at the middle of the post and at the cervical third of the root compared to the other types of reconstruction, irrespective of the presence of ferrule. These excessive concentrations of stress have been previously reported in the literature and have been associated with an increased risk of catastrophic root fracture requiring tooth avulsion [9, 26]. Again, as previously reported, MIC was also associated with higher ESV at the interface between root and cement, which could lead to CRR debonding and increased risk of failure [19, 26].

In our FEA, mFRC and mFRCG presented the lowest ESV on the root as compared to the other types of reconstruction in nonferrule models. This suggests that, in the absence of ferrule, mFRC could be an alternative to other types of reconstruction. Moreover, mFRC was designed to reduce root canal preparation. In the current FEA, similar root canal preparations were modeled to enable a valid comparison between all models. Another FEA is now required to evaluate whether a less invasive root canal preparation for mFRC would further reduce stress and risk of root fracture. Herein, addition of a gutta point in the bundle of fibers of mFRC did not increase the risk of fracture. This gutta point was intended to facilitate reintervention but has yet to be fully evaluated* in vitro* with tests of root canal retreatment on teeth reconstructed with mFRCG.

Concerning the reconstruction itself, the ROF of the CRR in mFRC was lower than that in sFRC, particularly in the nonferrule model, suggesting that peripheral fibers enabled reinforcement of the CRR; the hypothesis is that, even if peripheral fibers were to break following excessive stress, all the fibers and the sealing composite would have to be broken to completely fracture the CRR. This has to be confirmed by a mechanical study of mFRC resistance but is in agreement with previous papers. For instance, an oval fiber post system was shown to provide better stress distribution on the CRR and the root than a circular fiber post system, suggesting that fibers should be located on more peripheral parts of the root canal and not only in its center [28]. In addition, posts having the mechanical properties of both the fibers and the composite also present a better stress distribution than a post with only the mechanical properties of the fibers, suggesting that CRR with mFRC present better mechanical behavior [26]. This is also illustrated by a recent study that found that nanofibers enhanced the mechanical performance of dental restorative composite [32], increasing the number of biomedical applications [33]. Another point to consider is then the orientation of the fibers, as Vallittu et al. reported an anisotropic behavior of fiber-reinforced composite [34]. Different mechanical properties of CRR and root may therefore be obtained according to the orientation of fibers in the mFRC [34]. This could be tested in future FEA, and it would also be interesting to explore the number of fibers composing the CRR as this does not seem to have been evaluated in the literature.

## 5. Conclusion

Taken together, mFRC and mFRCG appear to be valuable alternatives for CRR to be used in a premolar with ferrule but particularly in the absence of ferrule. Mechanical and clinical studies are now necessary to evaluate these two CRR.

## Figures and Tables

**Table 1:** Mechanical properties of the homogeneous isotropic materials of the model.

Material	Elastic modulus (GPa)	Poisson's ratio	Tensile strength (MPa)	Reference No.
Zirconia crown	200	0.26		[19]

Composite resin	8.3	0.28	55	[19, 25]

Fiber glass post	40	0.27	99	[19, 25]

Sealing cement	0.1	0.20	3	[19, 25]

Metal post	110	0.32	145	[19, 25]

Dentin root	18.6	0.31	104	[19, 25]

Gutta-percha	0.00069	0.45		[9]

**Table 2:** Extreme stress values of the different parts of ferrule and nonferrule models.

	MIC (MPa)	sFRC (MPa)	mFRC (MPa)	mFRCG (MPa)
With ferrule				
CRR	55.4	5.6	14.7	13.9
Interface	26.3	8.7	12.9	12.7
Root	101.4	100.6	100.8	103.1
Without ferrule				
CRR	57.4	4.2	45.3	42.2
Interface	21.7	9.1	8.6	11.8
Root	134.5	156.2	130.0	131.2

Extreme stress values (ESV) are expressed in MPa for MIC (metal inlay core), single-fiber-reinforced composite (sFRC), multifiber-reinforced composite (mFRC), and multifiber-reinforced composite with gutta (mFRCG) for ferrule and nonferrule models.

**Table 3:** Risk of fracture of different parts of ferrule and nonferrule models.

	MIC	sFRC	mFRC	mFRCG
With ferrule				
CRR	0.38	0.37	0.15	0.14
Interface	8.76	0.58	0.90	0.84
Root	0.98	0.96	0.96	0.99
Without ferrule				
CRR	0.40	2.80	0.45	0.42
Interface	7.23	0.61	0.57	0.79
Root	1.29	1.50	1.25	1.26

The risk of fracture is expressed for MIC (metal inlay core), single-fiber-reinforced composite (sFRC), multifiber-reinforced composite (mFRC), and multifiber-reinforced composite with gutta (mFRCG) for ferrule and nonferrule models.

## Data Availability

The datasets generated and/or analyzed during the current study are available from the corresponding author upon reasonable request.
